# Exploring class III cellobiose dehydrogenase: sequence analysis and optimized recombinant expression

**DOI:** 10.1186/s12934-024-02420-2

**Published:** 2024-05-23

**Authors:** Angela Giorgianni, Alice Zenone, Leander Sützl, Florian Csarman, Roland Ludwig

**Affiliations:** Department of Food Science and Technology, Institute of Food Technology, BOKU University, Muthgasse 18, Vienna, 1190 Austria

**Keywords:** Cellobiose dehydrogenase, *Fusarium solani*, Hemoflavoenzyme, Heterologous expression, *Komagataella Phaffii*, Signal peptide shuffling

## Abstract

**Background:**

Cellobiose dehydrogenase (CDH) is an extracellular fungal oxidoreductase with multiple functions in plant biomass degradation. Its primary function as an auxiliary enzyme of lytic polysaccharide monooxygenase (LPMO) facilitates the efficient depolymerization of cellulose, hemicelluloses and other carbohydrate-based polymers. The synergistic action of CDH and LPMO that supports biomass-degrading hydrolases holds significant promise to harness renewable resources for the production of biofuels, chemicals, and modified materials in an environmentally sustainable manner. While previous phylogenetic analyses have identified four distinct classes of CDHs, only class I and II have been biochemically characterized so far.

**Results:**

Following a comprehensive database search aimed at identifying CDH sequences belonging to the so far uncharacterized class III for subsequent expression and biochemical characterization, we have curated an extensive compilation of putative CDH amino acid sequences. A sequence similarity network analysis was used to cluster them into the four distinct CDH classes. A total of 1237 sequences encoding putative class III CDHs were extracted from the network and used for phylogenetic analyses. The obtained phylogenetic tree was used to guide the selection of 11 *cdhIII* genes for recombinant expression in *Komagataella phaffii*. A small-scale expression screening procedure identified a promising *cdhIII* gene originating from the plant pathogen *Fusarium solani* (*Fs*CDH), which was selected for expression optimization by signal peptide shuffling and subsequent production in a 5-L bioreactor. The purified *Fs*CDH exhibits a UV-Vis spectrum and enzymatic activity similar to other characterized CDH classes.

**Conclusion:**

The successful production and functional characterization of *Fs*CDH proved that class III CDHs are catalytical active enzymes resembling the key properties of class I and class II CDHs. A detailed biochemical characterization based on the established expression and purification strategy can provide new insights into the evolutionary process shaping CDHs and leading to their differentiation into the four distinct classes. The findings have the potential to broaden our understanding of the biocatalytic application of CDH and LPMO for the oxidative depolymerization of polysaccharides.

**Supplementary Information:**

The online version contains supplementary material available at 10.1186/s12934-024-02420-2.

## Background

Cellobiose dehydrogenase (CDH, EC 1.1.99.18, CAZy auxiliary activity family AA3_1) is a hemoflavoenzyme produced by a variety of biomass-degrading fungi [1, 2]. Phylogenetic analyses revealed that CDH has evolved into four distinct classes [3–5]. While class I and II CDHs are well characterized and their roles in cellulose degradation are known, the catalytic function and substrate preferences of the other two classes are still unknown, highlighting a gap in our current understanding of the cellulolytic and enzymatic machinery employed by fungi. Due to this lack of information, the expression and characterization of CDH sequences from uncharacterized classes is a subject of ongoing research [6].

The first CDH (belonging to class I) was identified in 1974 in the secretome of the white-rot fungus *Phanerochaete chrysoporium* [7]. Since then, a number of studies investigated the catalytic and biochemical properties of CDHs, as well as their potential applications [8], including the development of biosensors for the detection of carbohydrates, such as lactose [9] or glucose [10], andthe production of biofuel cells [11]. CDH mainly exhibits a two-domain structure consisting of a heme *b*-containing N-terminal cytochrome (Cyt) domain and a catalytic FAD-dependent dehydrogenase (DH) domain belonging to the glucose-methanol-choline (GMC) superfamily [6, 12–14]. However, the Cyt domain may be absent in some sequences belonging to class I, II, and III, and putative CDH sequences belonging to the evolutionarily more distant class IV, which lack a Cyt domain entirely [5]. These findings suggest that this domain may not be essential for all functions of CDH in fungi, but it remains crucial for its electron transfer capacity [15].

In addition to the DH and Cyt domains, other structural features have also been identified to influence the functionality of the enzyme. Firstly, the Cyt and DH domains are connected by a flexible linker region. The length and composition of this region varies among the different CDH classes. Class I CDHs typically have a shorter and more conserved linker, consisting of 30–40 residues, while class II is characterized by a longer and more variable linker, usually between 40 and 50 residues [17]. Additional important structural features are found in the C-terminal region of CDHs. Carbohydrate-binding modules (CBM) are common in class II CDHs, leading to the further sub-classification of class IIA and IIB, depending on the presence or absence of this module, respectively. In a previous study, it was observed that 61% of the analyzed Class III CDH sequences have an additional C-terminal extension. However, only a small subset (6%) can be identified as CBMs. The function of the C-terminal extensions found in 55% of the sequences is unknown [5].

The phylogenetic differentiation of CDH sequences into initially two distinct classes was first proposed in 2004 by Zámocký et al.: class I including CDHs from basidiomycetes and class II consisting of ascomycetous CDHs [3]. The hypothesis of an additional putative class was then suggested in 2008, when a clear differentiation of three separate clades in a phylogenetic tree of CDH was observed [16]. Noteworthy, the putative CDH sequence from *Aspergillus fumigatus* was identified as an outlier, clustering apart from class I and II already in 2004. Subsequent research later identified this sequence as member of class III. Following more comprehensive phylogenetic analyses and support by the ever-growing number of available sequences, class III was officially recognized as a distinct class in 2011 [4], followed by the introduction of class IV in 2019 [5]. All class III and class IV CDH sequences known to date have been found among ascomycetous species.

So far, the biological role of CDH class III and IV remains enigmatic. Nonetheless, transcriptomic data indicate that fungi are actively producing CDH III, indicating their physiological relevance within the enzymatic machinery of filamentous fungi [18–21]. This observation suggests that the existence of CDHs III goes beyond non-expressed genetic information and that fungi actively transcribe and use *cdh III* genes.

The catalytic mechanism of the DH domain of known CDHs involves two half reactions. In the reductive half reaction, class I and II CDHs catalyze the oxidation of the anomeric C1 carbon of cellobiose to cellobiono-δ-lactone, which subsequently hydrolyzes to cellobionic acid. During the subsequent oxidative half-reaction, electrons are transferred from the reduced FADH_2_ in sequential single-electron transfer steps to the higher redox potential heme *b* cofactor of the Cyt domain, which acts as a mediating moiety to terminal electron acceptors [6]. The interdomain electron transfer (IET) between FAD and heme *b*, which occurs in the presence of a suitable electron donor like cellobiose, is considered the rate limiting step of CDH activity and shows different pH optima of class I and II CDHs [22, 23]. Class I CDHs typically show acidic pH optima for IET, but among class II CDHs IET happens at a wider range of pH, up to the neutral or even slightly alkaline range [6]. As an alternative to the electron transfer via the Cyt domain, electrons can also be transferred directly from the DH domain to external electron acceptors including quinones and molecular oxygen, producing hydroquinones and H_2_O_2_, respectively [4, 6, 15, 24].

In addition to the efficient conversion of cellobiose, CDHs are able to oxidize longer oligosaccharides resulting from cellulose degradation and activity has been found with a variety of other sugars such as glucose, lactose, and maltose. Unlike other GMC-oxidoreductases, CDH distinctly prefers disaccharides and oligosaccharides over monosaccharides [11]. Interestingly, ascomycetous CDHs, belonging to class II CDH, have been shown to be active on a broader substrate spectrum beside cellobiose compared to class I CDHs. Recently an enzyme belonging to this class could be characterized with a distinct preference for xylo-oligosaccharides [25].

Beside quinones and molecular oxygen, CDH can transfer electrons to a number of molecules with spectral properties useful for measuring enzymatic activity, such as ferrocene, cytochrome *c*, and 2,6-dichloroindophenol (DCIP) [6, 15]. However, recent research suggests the native electron acceptor of CDH is lytic polysaccharide monooxygenase (LPMO) and CDH primarily serves as a catalytic activator of LPMO in fungal secretomes. This synergy has been shown to boost cellulose degradation and enhances the overall degradation process [14, 26, 27]. To utilize these new findings for the efficient conversion of plant biomass, it is essential to understand the mechanisms behind biomass degradation [28, 29]. The molecular and catalytic characteristics of CDHs have been shown to be interconnected with the properties of the LPMOs they interact with in their natural environment [14]. CDHs have likely evolved to transform the products of polysaccharide degradation, generated by LPMOs, to serve as auxiliary support for the function of LPMO. The co-evolution between CDH and LPMO implies that the properties of distinct CDH classes may vary as a result of diverse adaptations.

Despite the substantial time that has passed since the initial discovery of class III within the CDH sequence diversity [16], to the best of our knowledge, no reports appear available on the successful production of an active member belonging to this class of enzymes. The reasons for the challenges in finding an effective expression strategy for this class of enzymes remain unclear. The aim of this study is to conduct a comprehensive phylogenetic analysis of CDH sequences to identify CDH III members for recombinant expression. The subsequent investigation of the biochemical and catalytic properties of these enzymes targets to further explore diversity among different CDH classes and thereby contributing to a broader understanding of the enzymes present in fungal secretomes.

## Results and discussion

### Database mining of CDH sequences

To select representative class III CDH sequences for subsequent expression and characterization, we compiled an updated list of all available sequences from two databases, employing two different search strategies. The first search was conducted on NCBI using BLAST and 14 to date biochemically characterized class I and II CDH sequences that served as individual queries (Additional file 1: Table S1). The results of the individual searches were merged and duplicated sequences were deleted, resulting in 3323 sequences. The second search was performed on UniProt using the profile hidden Markov Models search tool hmmsearch and an alignment of the same 14 aforementioned characterized CDH sequences as query. The search yielded 2046 sequences. To ascertain that the majority of hits would correspond only to CDH sequences, both searches were restricted to fungal organisms and employed an e-value cut-off that excluded most sequences of other GMC family members, as known from previous analysis [5]. The sequences of the two search results were combined with the 14 characterized CDHs and 17 additional characterized non-CDH enzymes belonging to the GMC family, which served as outgroups (Additional file 1: Table S2, Additional file 2).

### Sequence similarity network analysis

Due to the high number of sequences in the final dataset, employing conventional phylogenetic analysis with typical tree-building algorithms proved impractical for clustering sequences into the previously identified CDH classes [3–5, 16]. To address this issue, we adopted an alternative strategy by constructing a sequence similarity network (SSN) for subsequent analyses.

**Fig. 1:**
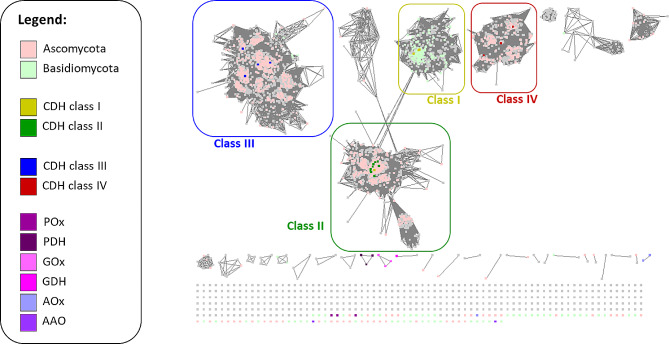
Sequence similarity network for CDH sequences obtained from NCBI and UniProt database searches at an AS cut-off of 10^− 160^. Sequences originating from Ascomycota (pink) and Basidiomycota (green) are indicated whenever taxonomic information was available. Members of previously identified CDH classes have been additionally highlighted. Characterized non-CDH GMC enzymes are indicated in various colors according to the legend (pyranose oxidase POx; pyranose dehydrogenase PDH; glucose oxidase GOx; glucose dehydrogenase GDH; alcohol oxidase AOx; aryl-alcohol oxidoreductase AAO)

Within the network, the alignment score (AS) was utilized to measure the similarity between sequence pairs, where a lower AS indicates stronger similarity. The resulting network was studied at a series of decreasing AS cut-offs to follow the formation of separated sequence clusters. Notably, the 17 characterized non-CDH GMC enzymes, serving as outgroups, already separated from the cluster of CDH sequences at an AS cut-off of 10^− 40^. CDH class IV, the most distant class of CDHs, separated at 10^− 100^, followed by CDH class III at 10^− 140^. Finally, at 10^− 160^, we could observe a spatial separation of basidiomycetous class I and ascomycetous class II sequences in the SSN (Fig. 1, Additional file 1: Fig. S1 to S3). The early divergence of the 17 outgroup GMC enzymes, together with the fact that no major cluster besides the CDH clusters was found, affirms that the applied search criteria resulted predominantly in CDH sequences. In order to study the sequences of the CDH class III cluster in more detail, we extracted its 1237 sequences from the SSN and subjected them to further analysis.

### Sequence and phylogenetic analysis

To select sequences that are suitable for expression, we applied multiple criteria to eliminate potentially non-functional candidates. Firstly, sequences were only considered for selection if their sequence length was between 700 and 1000 residues, indicating a full-length CDH (Additional file 3). Moreover, sequences were analyzed for the presence of a pre-pro leader sequence. Any sequences lacking this signal peptide were not considered to be extracellular CDHs and were excluded. Additionally, we ensured the presence of the following key features in the sequences: (i) FAD-binding motif (Rossmann fold consensus sequence, GxGxxG); (ii) catalytic His in the active site of the DH domain (His689 in CDH from *Phanerochaete chrysosporium* (*Pc*CDH)); (iii) presence of a Cyt domain and (iv) heme coordinating residues Met and His in the Cyt domain (Met65 and His163 in *Pc*CDH). Sequences lacking these motifs or residues were excluded from the sequence selection, leading to a total of 857 sequences.

**Fig. 2:**
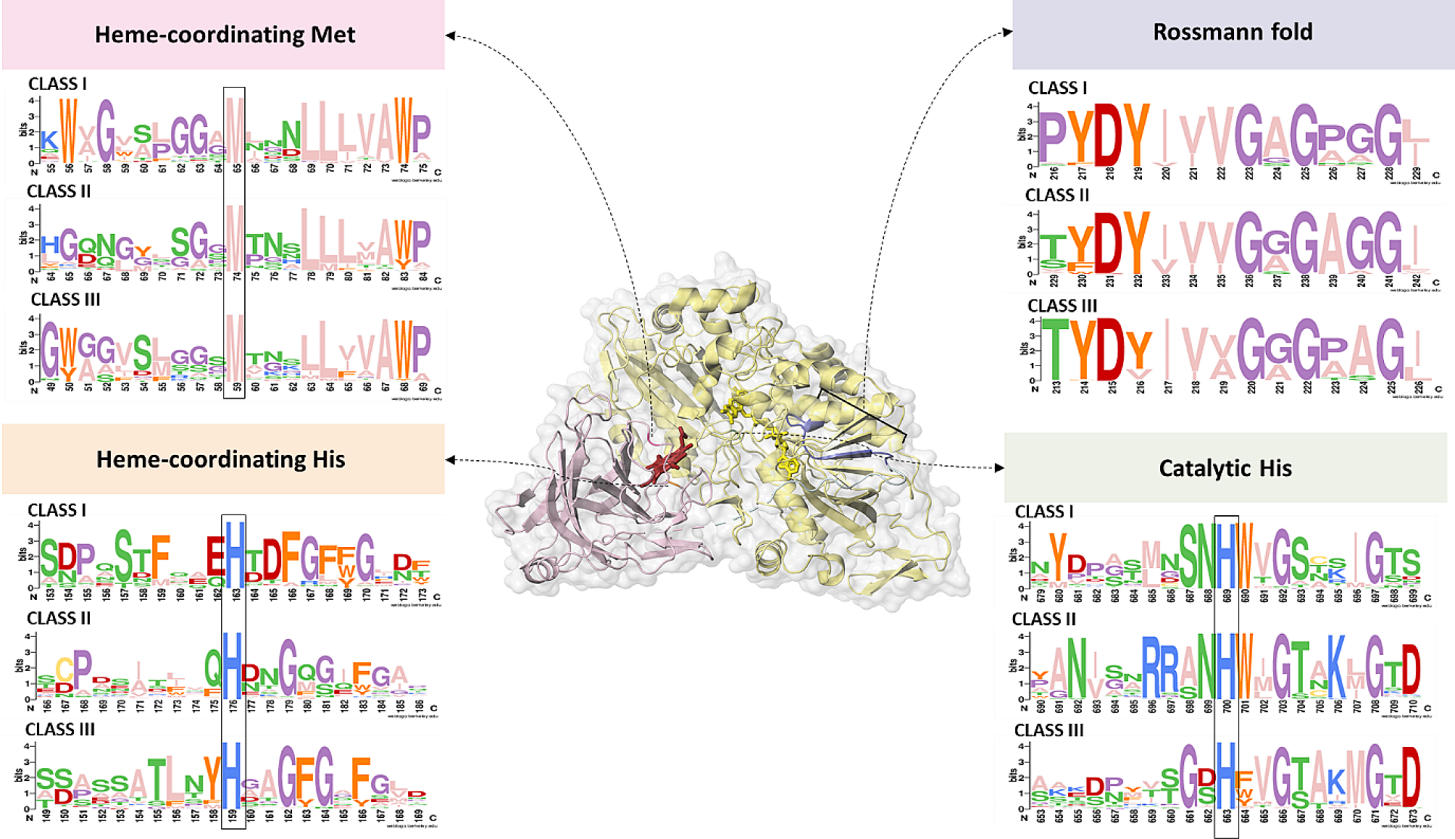
Comparison of sequence logos for conserved regions or catalytical residues of CDH classes I, II and III. The specific positions of these amino acid residues are indicated within the structure of CDH from *Thermothelomyces myriococcoides* (PDB: 4QI6, syn. *Myriococcum thermophilum*)

Analysis of sequence logos for regions around the mentioned key features revealed a strict conservation of key residues across CDH classes I, II and III (Fig. 2). Notably, the logos did not reveal any clear specific differences between the three classes. The only exception is found in the direct vicinity of the catalytic His. In particular, the two positions before the catalytic His (Ser687 and Asn688 in *Pc*CDH), which were shown to form hydrogen bonds with the substrate in *Pc*CDH [24], exhibit variations across the three CDH classes. Highly conserved Ser and Asn residues are found in class I sequences, a less conserved Ala/Ser and highly conserved Asn in class II sequences and a highly conserved Gly and less conserved Asp/Ser in sequences belonging to class III. For class I CDHs, structural analysis of *Pc*CDH has revealed that Ser687 forms a hydrogen bond between the main polypeptide chain and the substrate. This suggests that a different amino acid side chain at this position does not significantly influence substrate binding or alter substrate specificity. Only the adjacent Asn residue, that is absent in class III, has its side chain actively participating in the hydrogen bonding of the substrate. Variations in this position, observed in class III in contrast to the strict conservation in classes I and II, could therefore potentially cause differences in the substrate specificity and catalytic properties.

To reduce redundancy in the sequence selection of CDH III for phylogenetic analysis, only a single representative sequence was preserved for all sequences displaying 99% sequence identity or above, resulting in a final selection of 367 sequences. Finally, known members of CDH class I and II were included in the selection to act as outgroups in the phylogenetic tree of CDH class III sequences (Additional file 4).

**Fig. 3:**
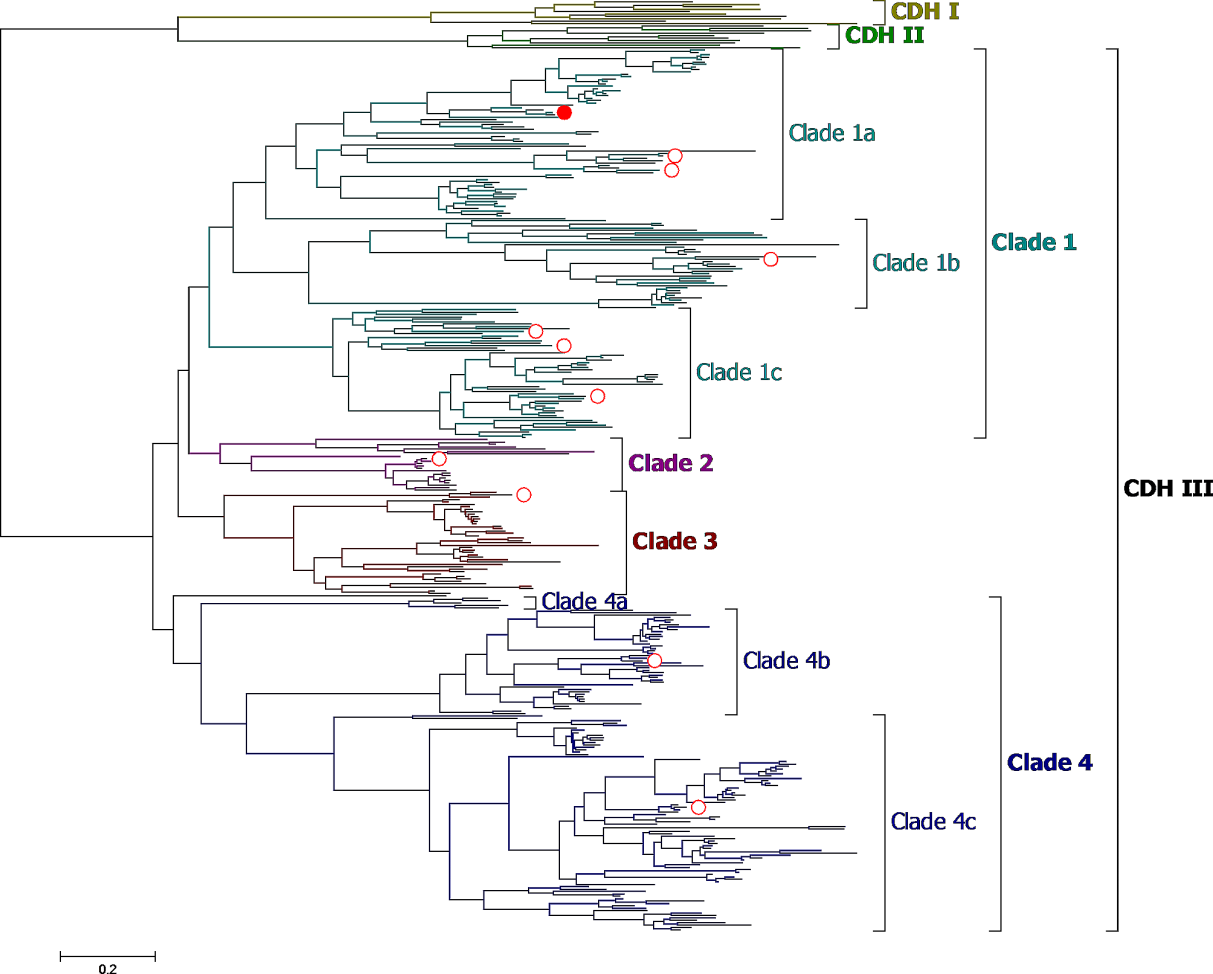
A maximum likelihood phylogenetic tree was generated based on 387 CDH sequences (10 CDH I, 10 CDH II, 367 CDH III sequences) and reveals distinct sub-clades for CDH class III. Sequences selected for expression in this study are indicated by a red circle. The full red circle marks the sequence from *Fusarium solani* (KAH7237211.1). Characterized class I (ochre) and II (green) sequences served as outgroups

The inferred maximum likelihood phylogenetic tree shows a clear separation between the outgroups (class I and II CDH) and class III CDH (Fig. 3, Additional file 5), which itself was further separated into sub-clades defined by an evolutionary distance threshold of 0.4. Based on the tree, a set of 11 class III sequences was chosen for expression, ensuring a good distribution across all sub-clades (Fig. 3, Additional file 1: Table S3). For the selection we prioritized sequences from organisms with plant-associated lifestyles, such as plant pathogenic fungi or endophytes.

### Cloning of CDH III sequences via modular assembly and yeast integration

To maintain a high flexibility for the subsequent cloning of different *cdhIII* genes, we made use of a modular cloning technique based on golden gate cloning [30] for the precise and efficient assembly of DNA fragments. The selected genes were obtained, whenever possible, as separate synthetic linear fragments, coding for the signal peptide (SP), the Cyt domain, or the DH domain, with appropriate overhangs for subsequent cloning. The linear cDNA fragments were incorporated into a cloning vector, and resulting donor plasmids were used for *E. coli* transformation with a spectinomycin resistance and blue-white colony selection system to identify recombinant clones. Donor plasmids containing distinct gene modules (SP, Cyt, DH or C-terminal-tag) were purified, mixed, and used for the insertion into an acceptor plasmid with either GAP or AOX promoter, respectively, in two separated one-pot reactions. The correct integration into the plasmid was verified by DNA sequencing. Following linearization, the final integration plasmid was used for transformation into *Komagataella phaffii*, and multiple hygromycin-resistant clones for each of the genes were selected for subsequent experiments.

### Small-scale expression identified promising CDH III sequences

A preliminary screening for the identification of CDH III-expressing clones was performed using yeast transformants harboring the gene of interest under the control of the constitutive GAP promoter. This allowed for simpler culturing conditions compared to the inducible AOX promoter system. Single clones were grown in 250 ml YPD media for six days with the addition of 2% glucose as feed three days after the inoculation. To confirm the production and secretion of the protein of interest, SDS-PAGE analysis was performed using the culture supernatant after deglycosylation with Endo H_f_ (NEB). (Additional file 1, Fig. S4). A distinct band with an apparent molecular weight of approximately 100 kDa was observed only for the clones transformed with the *cdhIII* gene from *Fusarium solani* (ID KAH7237211.1). This gene was therefore selected as candidate for further expression studies.

### Optimizing protein expression: Signal peptide shuffling and testing of different promoters

**Fig. 4:**
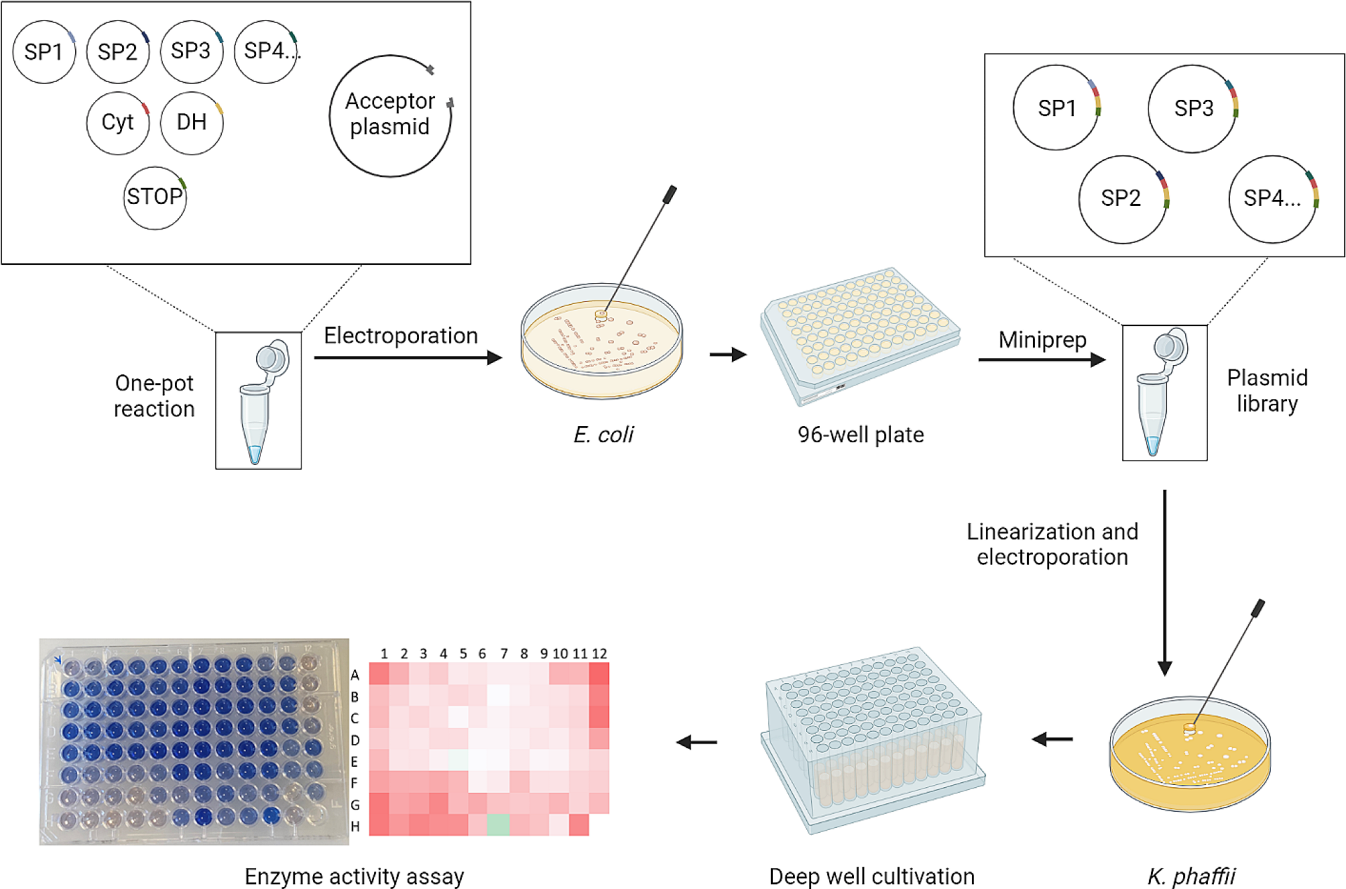
Signal peptide shuffling workflow. A one-pot reaction in which donor plasmids harboring the different gene modules are mixed at equal ratios with an acceptor plasmid. After *E. coli* transformation and selecting clones for the cultivation in a 96-well plate, a plasmid library is generated and transformed into *K. phaffii*. Transformants are selected and cultivated in deep-well-plates. The culture supernatant is used for activity assays and colonies showing the highest enzyme activities are selected for further analyses. Figure created with BioRender.com.

To enhance expression yield and to identify the best recombinant yeast clone, we implemented a dual approach involving a signal peptide shuffling strategy along with the evaluation of different promoter systems [31, 32]. The cloning procedure was conducted in one-pot reactions containing donor plasmids carrying sequences for 10 different signal peptides, the gene of interest and the C-terminal STOP-Tag, respectively, all at the same molar ratio, alongside a selected acceptor plasmid for the integration of the resulting gene construct. (Fig. 4). Two different acceptor plasmids were used in two separated reactions, one featuring the constitutive GAP promoter and the other containing the methanol-induced AOX promoter. After the cloning procedure in *E. coli*, we used the two isolated plasmid libraries, containing the *cdhIII* gene with 10 different signal peptide sequences (Additional file 1: Table S4), to transform *K. phaffii*. We selected 96 successful yeast transformants obtained for each of the two promoters (GAP and AOX) and screened them for CDH production by cultivating the clones in deep well plates. Heterologous production was induced in the AOX plate by adding methanol-containing medium 3 days after inoculation in BYPD medium, followed by regular feeding with methanol, until the supernatant was harvested on day 6. The GAP-promoter-dependent culture did not require specific inducing conditions. Expression tests were performed in BYPD medium, with glucose as the sole carbon source and no additional feeding, with the supernatant harvested 3 days after inoculation.

The culture supernatant resulting from the plate expression was analyzed for CDH activity using the standard DCIP assay with cellobiose as substrate at pH 6.0. From the clones with the methanol-inducible AOX promoter, 69% showed an activity higher than the limit of quantification of the assay, whereas no clone transformed with the GAP-promoter-containing construct produced enzyme activity higher than this limit. While the production of *cdh* genes under the control of AOX promoter has been well documented [33–35], there is only limited available data regarding CDH expression under the control of the GAP promoter [36], despite multiple reports on the use of this expression system for various other lignocellulolytic enzymes [37–42]. Our analysis shows that the expression system utilizing the AOX promoter was more successful than the GAP promoter to obtain detectable activities of *Fs*CDH. The ten clones resulting in the highest enzyme activities were further investigated by colony PCR to verify the gene encoding for *Fs*CDH and to identify the associated signal peptide. Intriguingly, four of them contained the native signal peptide of the *Fs*CDH sequence, three contained the signal peptide of a second CDH III sequence present in *F. solani* (*Fs*CDH2, ID KAH7254564.1) and three more contained the well-characterized α-mating factor from *S. cerevisiae*. Based on the results obtained within this screening of the most promising clone, harboring *Fs*CDH downstream of the *F. solani* native signal peptide and the AOX promoter, was selected for additional protein expression experiments.

### Production and purification of *Fs*CDH

**Fig. 5:**
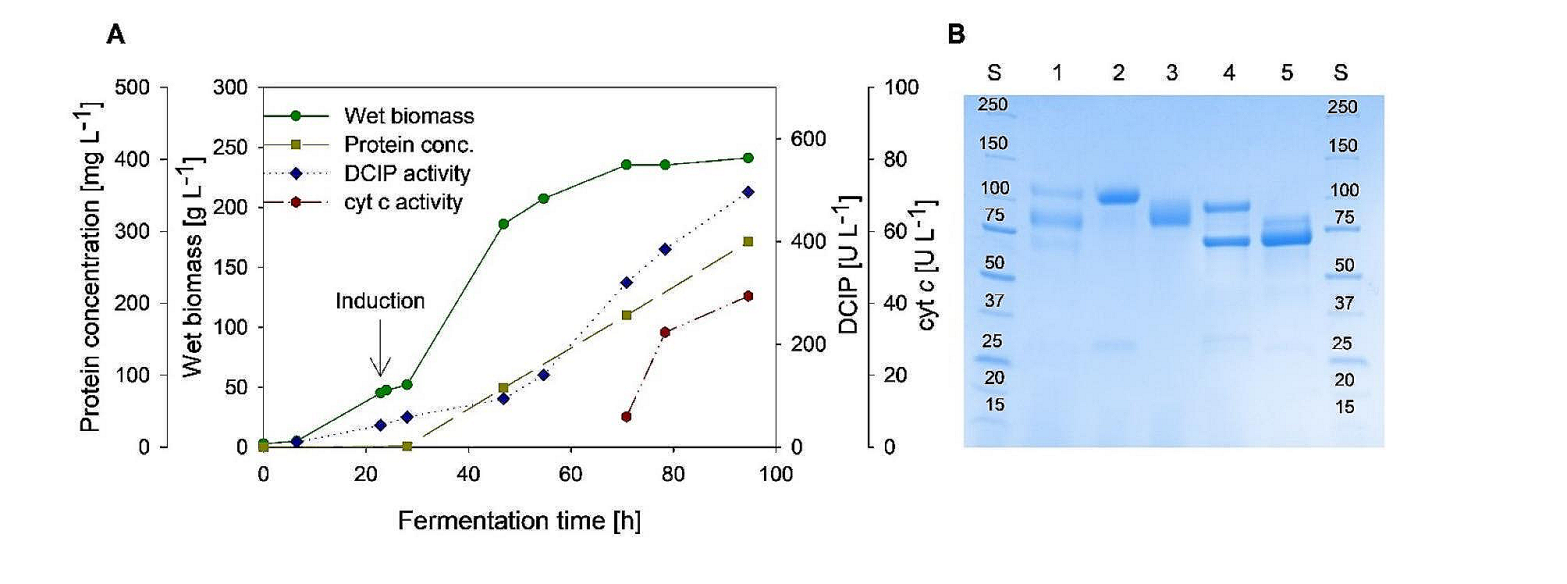
(**A**) Heterologous expression of *Fs*CDH in a 5-L bioreactor monitoring wet biomass (green circle), protein concentration determined by the Bradford assay (yellow square), enzymatic activity measured with DCIP (blue diamond) and cytochrome *c* as electron acceptor (red hexagons). (**B**) Coomassie blue stained SDS-PAGE results of samples of *Fs*CDH before and after deglycosylation with Endo H_f_. The Precision Plus Protein Dual Color Standard (Bio-Rad) is shown in Lane S. Lane 1 shows harvest sample taken at the end of fermentation. Lane 2 and 4 show purified full-length *Fs*CDH before and after deglycosylation, respectively. Lane 3 and 5 show the isolated dehydrogenase domain *Fs*DH before and after deglycosylation, respectively. Endo H_f_ is present in Lanes 4 and 5 showing a single additional band at approximately 70 kDa

To produce a sufficient amount of enzyme for further characterization, the previously identified yeast clone was used for heterologous production of *Fs*CDH in a 5-L bioreactor following a fed-batch protocol as described by Pichia Fermentation Process guidelines of Invitrogen [43]. After an initial batch and fed-batch phase using glycerol as the carbon source, *Fs*CDH expression was induced by the addition of methanol approximately 24 h after inoculation. At the end of the fermentation process, the total *Fs*CDH production reached 497 U L^− 1^, measured with DCIP as electron acceptor at pH 6.0 after 95 h from the inoculation and approximately 71 h of induction (Fig. 5A). Class I and class II CDHs, produced in the same expression host, have been reported to achieve higher volumetric activities of 1800 U L^− 1^ for CDH from *Phanerochaete chrysosporium* (*Pc*CDH) [33] and 1700 U L^− 1^ for CDH from *Neurospora crassa* (*Nc*CDHIIA) [44]. However, the activity reached depends strongly on the CDH gene studied. For instance, for the production of another class II CDH from *N. crassa* (*Nc*CDHIIB), following the same protocol, a volumetric activity of only 410 U L^− 1^ is reported [44]. These values clearly show the gene-dependent variations in expression levels of CDH using *K. phaffii*.

To obtain a homogeneous enzyme solution for further characterization, a two-step purification strategy was implemented. The first step involved hydrophobic interaction chromatography (HIC), and the second step anionic exchange chromatography (AEX), as reported for other CDHs [34, 35]. The homogeneity of the resulting enzyme preparation was confirmed by determining the RZ value (Table 1) from UV-Vis spectra and by SDS-PAGE (Fig. 5B). During the purification process, two fractions of the enzyme were collected: a red fraction, consisting of the full-length *Fs*CDH, and a yellow fraction, consisting of the DH domain (*Fs*DH) only, missing the cleaved Cyt domain. This phenomenon has been consistently observed in both homologous and heterologous production of CDH. The occurrence is primarily attributed to the linker region within the CDH structure, which is susceptible to proteolytic cleavage [44, 45].

The identity of the purified enzyme preparations was verified by LC-ESI-MS and the molar masses of both *Fs*CDH and *Fs*DH were determined by SDS-PAGE analysis (Fig. 5B), both, before and after treatment with Endo H_f_ for deglycosylation. *Fs*CDH exhibited a molar mass of 99.0 kDa, which decreased to 90.6 kDa after deglycosylation. *Fs*DH displayed a molar mass of 82.5 kDa before deglycosylation, which decreased to 78.5 kDa after treatment with Endo H_f_.

Enzymatic activity of the obtained two fractions was evaluated during the purification procedure. The purified full-length *Fs*CDH exhibited a specific activity of 1.64 U mg^− 1^ in the DCIP assay and 0.11 U mg^− 1^ with cytochrome *c* as the electron acceptor. In comparison, *Fs*DH showed a specific activity of 2.18 U mg^− 1^ using the DCIP assay and no detectable activity in the cytochrome *c* assay, as expected, since *Fs*DH is lacking the Cyt domain. The difference in specific activity observed for the DCIP assay, which is higher for *Fs*DH, is likely due to the difference in molecular weight of the two enzymes.

**Table 1:** Purification tables for heterologous production of *Fs*CDH (A) for full-length *Fs*CDH and (B) for *Fs*DH.

**A) CDH**										
				**DCIP**		**cytochrome ** *c*				
	**Vol. [ml]**	**Prot. conc. ** **[mg mL**^− 1^]	**Total protein [mg]**	**Total act.** ^c^ ** [U]**	**Specific act.**^c^** [U mg**^− 1^]	**Total act.** ^d^ ** [U]**	**Specific act.**^d^** [U mg**^− 1^]	**RZ A**_420_/**A**_280_	**Yield [%]**	**Purif. factor [fold]**
**Supernat.**	4500	0.3^a^	1310	946	0.72	47	0.04	-	100	1
**HIC (CDH)**	750	0.8^a^	598	542	0.91	14	0.02	0.33	57	1.3
**AEX (CDH)**	15	4.5^a^	68	111	1.64	8	0.11	0.53	12	2.3
		9.9^b^	149							
**B) DH**										
				**DCIP**		**cytochrome ** *c*				
	**Vol. [ml]**	**Prot.** **conc. ** **[mg mL**^− 1^]	**Total protein [mg]**	**Total act.** ^**c**^ **[U]**	**Specific act.** ^**c**^ **[U mg** ^**− 1**^ **]**	**Total act.** ^**d**^ **[U]**	**Specific act.** ^**d**^ **[U mg** ^**− 1**^ **]**	**RZ A**_420_/A_280_	**Yield [%]**	**Purif. factor [fold]**
**Supernat.**	4500	0.3^a^	1310	946	0.72	47	0.04	-	100	1
**HIC (DH)**	1800	0.6^a^	1120	860	0.77	b.d.l.	b.d.l.	0.12	91	1.1
**AEX (DH)**	30	6.1^a^	183	399	2.18	b.d.l.	b.d.l.	0.02	42	3.0
		11.8^b^	354							

b.d.l. = below detection limit

^a^ Protein concentration measured with the Bradford assay

^b^ Protein concentration calculated from the absorbance at 280 nm using an ε_280_ = 100,185 M^− 1^cm^− 1^ for CDH or an ε_280_ = 77,600 M^− 1^cm^− 1^ for the DH domain

^c^ Activity measured with the standard DCIP assay

^d^ Activity measured with the standard cytochrome *c* assay

### UV-Vis spectroscopy confirms typical CDH features in *Fs*CDH

**Fig. 6:**
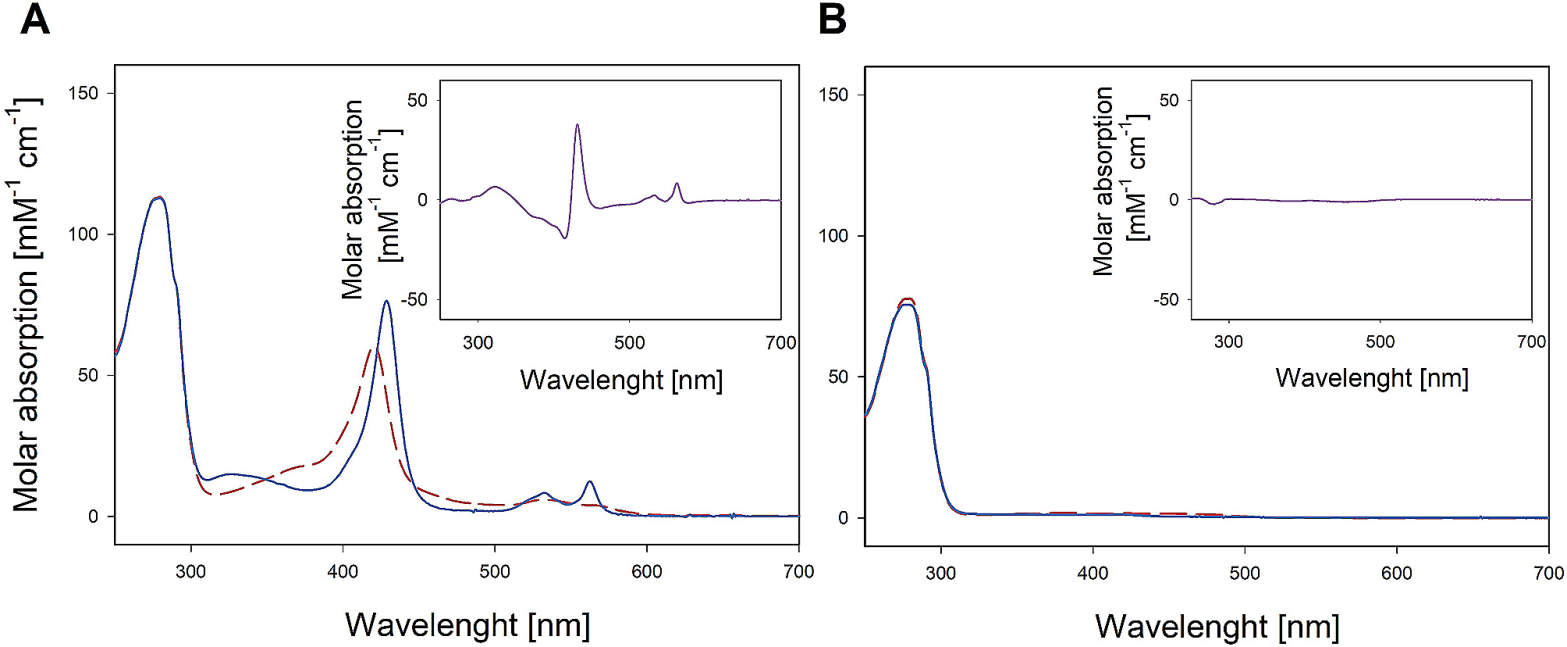
UV-Vis spectra recorded for **A)** full-length *Fs*CDH and **B) ***Fs*DH. Oxidized enzyme spectra are shown in red dashed line, while reduced spectra are shown as a blue solid line and differential spectra are shown as inserts. Reduction was obtained by addition of 1 mM cellobiose to the enzyme solution in 50 mM potassium phosphate buffer pH 6.0.Spectra were recorded after 10 min of incubation at 30 °C

The spectral properties of class III CDH were studied by recording UV-Vis spectra for both full-length *Fs*CDH and the isolated dehydrogenase domain *Fs*DH (Fig. 6). The results show a typical flavocytochrome spectrum for the full-length *Fs*CDH, with the dominant heme *b* Soret band found with a maximum at 420 nm. Upon the addition of cellobiose and reduction of the heme, this band shifted to 429 nm. Simultaneously, there is a significant increase in the intensity of the characteristic α- and β-bands. This is in accordance with previously observed spectral characteristics of characterized class I and II CDHs [34, 44]. In contrast to the full-length enzyme, *Fs*DH lacks these distinct features, due to the absence of the heme-containing Cyt domain. The FAD occupancy was calculated from the recorded spectra as described by Wohlschlager et al. [34]. The iterative calculation indicated 9% FAD loading for the full-length enzyme and 13% for the isolated DH domain. While these FAD occupancy percentages appear relatively low when compared to CDHs produced homologously (92–100%), or heterologously in *Trichoderma reseei* (70%) [34, 35], they align more closely with data reported for CDHs heterologously produced in *K. phaffii*, which typically exhibit FAD occupancies ranging from 30 to 40% [10, 35]. The reduced FAD loading could arise from difficulties in the post-translational protein processing within the foreign expression host or potential hyperglycosylation, which might impede proper folding and thereby lower FAD binding capacity.

### Exploring the catalytic properties of *Fs*CDH

To investigate the catalytic properties of *Fs*CDH, we performed two different activity measurements with the 2,6-dichloroindophenol (DCIP) and the cytochrome *c* assay (Table 2). The first method allowed us to determine the reduction of an electron acceptor by the transfer of electrons from the FAD-containing DH domain. The second assay involves the reduction of cytochrome *c* via the heme *b* of the Cyt domain. This is dependent on the IET between the flavin and the heme group within the two domains of *Fs*CDH. To cover a broader range of conditions, we tested three different pH values: pH 4.0, 6.0 and 8.0. The same assay, using the previously characterized class II CDH from *Thermothelomyces myriococcoides* (*Mt*CDH, syn. *Myriococcum thermophilum*), was performed for comparison and as a control of the experimental conditions. The results show a specific activity of *Fs*CDH for cellobiose of 0.8 U mg^− 1^ at pH 6.0, which decreases to 0.6 U mg^− 1^ at pH 4.0. No activity was detected at pH 8.0. The same trend is observed with lactose as a substrate. For the well-known *Mt*CDH the same tendency is observed. Also, for both enzymes the specific activities using cytochrome *c* instead of DCIP as an electron acceptor are generally lower and with cytochrome *c* the highest specific activity is obtained at pH 4. When compared to CDH activities reported in literature [15], it is obvious that *Fs*CDH has a lower specific activity for both the DCIP and cytochrome *c* assays. An explanation for this is the low FAD occupancy of the catalytic DH domain in the recombinantly expressed enzyme. Recalculating the specific activities for a 100% occupancy shows that *Fs*CDH is a CDH III with only slightly lower activity than the previously characterized *Mt*CDH (Table 3). However, both activities appear to be significantly lower when compared with published data from two other characterized CDHs from *Thermothelomyces fergusii* syn. *Corynascus thermophilus* (*Ct*CDH, class II) and *Pc*CDH, (class I).

**Table 2:** Analysis of *Fs*CDH and *Mt*CDH specific activities at different pH values

		*Fs*CDH						*Mt*CDH					
		Cellobiose			Lactose			Cellobiose			Lactose		
		pH 4	pH 6	pH 8	pH 4	pH 6	pH 8	pH 4	pH 6	pH 8	pH 4	pH 6	pH 8
**DCIP**	**Specific activity** **[U mg** ^**− 1**^ **]**	0.60 ± 0.07	0.82 ± 0.03	b.d.l.	0.61 ± 0.08	0.67 ± 0.20	0.05 ± 0.03	1.30 ± 0.02	1.47 ± 0.05	0.24 ± 0.01	1.31 ± 0.13	1.59 ± 0.32	0.17 ± 0.03
**cyt** ***c***	**Specific activity** **[U mg** ^**− 1**^ **]**	0.16 ± 0.01	0.04 ± 0.00	0.01 ± 0.01	0.19 ± 0.01	0.05 ± 0.00	b.d.l.	0.71 ± 0.04	0.22 ± 0.04	0.04 ± 0.00	0.84 ± 0.02	0.24 ± 0.02	0.04 ± 0.00

b.d.l.: below detection limit

Data is presented as mean values ± standard deviations

**Table 3:** Comparison of CDH specific activities and FAD occupancy

	Experimental conditions		Specific activity [U mg^− 1^]	FAD occupancy [%]	Specific activity (100% FAD loading) [U mg^− 1^]^a^	Reference
***Fs*** **CDH**	**pH**	6.0	0.7 ± 0.2	9%	7.4 ± 2.2	this publication
	**buffer**	50 mM KPB				
	**substrate**	lactose				
***Mt*** **CDH**	**pH**	6.0	1.6 ± 0.3	19%	8.4 ± 1.6	this publication
	**buffer**	50 mM KPB				
	**substrate**	lactose				
***Ct*** **CDH**	**pH**	5.5	9.4 ± 0.2	44%	21.4 ± 0.5	[35]
	**buffer**	50 mM NaAc				
	**substrate**	lactose				
***Pc*** **CDH**	**pH**	4.5	23.4 ± 1.0	70%	33.4 ± 1.4	[34]
	**buffer**	100 mM Britton-Robinson				
	**substrate**	lactose				

^a^: recalculated enzyme activity assuming 100% FAD loading

## Conclusions

In this study, we successfully achieved the recombinant expression of a class III CDH and verified its catalytic activity for the first time. To achieve the first active expression of a CDH III, an exhaustive collection of putative CDH III sequences was compiled through database mining and sequence similarity network analyses. This collection was instrumental in constructing a phylogenetic tree to ensure a representative selection from the extended sequence space of CDH class III sequences. From this set of selected sequences, the CDH from the ubiquitous plant-pathogenic ascomycete *F. solani* (*Fs*CDH) was successfully expressed in *K. phaffii* and the production was optimized by screening a combination of shuffled signal peptides with two different promoter systems. The activity of the enzyme with the typical CDH substrates cellobiose and lactose, as well as its ability to reduce the commonly used electron acceptors DCIP and cytochrome *c*, could be confirmed and is in line with the properties of known CDHs belonging to class I and class II.

However, the study revealed a low success rate for enzyme expression, with only one candidate successfully produced out of the eleven screened, and a notably low cofactor occupancy of the DH domain. These observations raise concerns about potential problems of the commonly used *K. phaffii* expression system or the typically chosen expression conditions for CDH III enzyme production. The obtained catalytic characteristics, which so far are similar to class I and class II CDHs, prompt the question of the evolutionary function of the substantial number of class III CDHs found within fungal genomes. An answer to this question will require the elucidation of differences between the CDH classes and will open the door for a deeper understanding of the biological function of CDHs. Further exploration might therefore involve investigating the evolutionary adaptations that led to the differentiation of various CDH classes or delving into the mechanisms underlying oxidative degradation of plant polysaccharides.

## Materials and methods

### Microbial strains, media and solutions

The chemicals used in this study were purchased with the highest purity available from Sigma, Fluka, Roth, or VWR. Primers were synthesized by Microsynth and their nucleotide sequences are provided in Additional file 1: Table S5. Restriction enzymes, dNTP mix and T4 DNA ligase were supplied by New England Biolabs (NEB). The plasmids pAGM9121, pPAP009 and pPAP010 (https://www.addgene.org/kits/marillonnet-yeast-secrete-detect/) were used for cloning in *E. coli*. Plasmids pPAP009 and pPAP010 are modifications of the pPAP003 vector from the Addgene kit mentioned above. They were used for gene expression in *K. phaffii* and they differ from pPAP003 in the use of distinct promoters for the expression of the gene of interest: the constitutive GAP promoter for pPAP009, or the inducible AOX promoter for pPAP010 (Additional file 1: Fig. S5). *E. coli* NEB 5α cells (NEB) were used for all cloning procedures and plasmid propagation, using the following culture conditions: 37 °C in LB (lysogeny broth) medium or on selective LB plates (containing 100 mg L^–1^ spectinomycin or 50 mg L^–1^ kanamycin). *K. phaffii* X-33 (Invitrogen) was used for extracellular expression of CDH III. Yeast cells were grown at 30 °C in BYPD broth medium consisting of 10 g L^− 1^ yeast extract, 20 g L^− 1^ peptone from casein, 4 g L^− 1^ dextrose, 100 mM potassium phosphate pH 6.0 or on YPD agar plates (10 g L^− 1^ yeast extract, 20 g L^− 1^ peptone from casein, 4 g L^− 1^ dextrose and 15 g L^− 1^ agar agar) containing 150 mg L^− 1^ hygromycin for selection of transformants. The fermentation medium was prepared in accordance with the Invitrogen Pichia Fermentation Process Guidelines [43]. *Mt*CDH from *Thermothelomyces myriococcoides* (syn. *Crassicarpon hotsonii*, syn. *Myriococcum thermophilum*) was produced as described before [46].

### Database mining and sequence similarity network

NCBI database searches were conducted using the protein BLAST algorithm (https://blast.ncbi.nlm.nih.gov/blast/Blast.cgi) [47] with 14 characterized CDHs, listed in Additional file 1: Table S1, as individual queries. The searches were limited to the kingdom of Fungi and an e-value cut-off of 10^− 30^. Subsequently, the results of all individual searches were combined, and duplicate sequence IDs were removed. The UniProt database search was performed using the online-tool hmmsearch (https://www.ebi.ac.uk/Tools/hmmer/) [48] and using an alignment of 14 characterized CDHs (Additional file 1: Table S1) as input. The search was limited to the kingdom of Fungi and an e-value cut-off of 10^− 30^. Sequence hits of both database searches (accessed on 27.01.2022) were combined and the 14 characterized CDHs were added to the sequence selection. Additionally, 17 characterized enzymes, belonging to the GMC superfamily, were added as outgroups (Additional file 1: Table S2). The resulting dataset, containing 5400 sequences (Additional file 2), was submitted to EFI-Enzyme Similarity Tool (https://efi.igb.illinois.edu/efi-est/) [49] to calculate a sequence similarity network. The network was finalized at an alignment score threshold of 10^− 30^ and a sequence length restriction between 150 and 1100 amino acids. Visualization of the SSN at different alignment score cut-offs was conducted in Cytoscape v3.9.1 [50].

### Phylogenetic analysis

All protein sequences of the SSN cluster containing putative CDH III sequences were extracted at an alignment score threshold of 10^− 160^. Sequences with identical IDs were removed and the dataset was restricted to a length between 700 and 1000 residues (Additional file 3). The dataset was further refined to exclude potentially non-functional sequences based on the following criteria: (i) sequences should show a signal peptide predicted by SignalP 5.0 (https://services.healthtech.dtu.dk/services/SignalP-5.0/) [51], (ii) contain the FAD-binding Rossmann fold consensus sequence motif (GxGxxG), (iii) show a catalytic His in the active site of the DH domain, (iv) contain a N-terminal Cyt domain, and (v) the Cyt domain should contain the heme coordinating residues Met and His. Identifying the essential residues and domains, that were used as selection criteria in putative CDH III sequences, was achieved by aligning the sequences together with the well-characterized sequence of class I CDH from *P. chrysosporium* (*Pc*CDH, PDB:1NAA) using MAFFT (G-INS-I algorithm) [52]. Sequences failing to meet one or more criteria described above were removed from the selection, bringing the total sequence count from 963 to 857.

Sequence redundancy was removed in Jalview (version 2.11.1.4), using a ≥ 99% sequence identity threshold [53], and 10 biochemically characterized sequences of CDH class I and 10 of CDH class II were added as outgroups, resulting in 390 sequences. Taxonomic annotation and removal of sequences containing non-proteinogenic characters was performed using the online tool SeqScrub (http://seqscrub.gabefoley.com/) [54].

The final dataset for phylogenetic analysis (10 CDH I + 10 CDH II + 367 CDH III, Additional file 4) was aligned by MAFFT (G-INS-i) and trimmed for positions with ≥ 90% gaps using TrimAl 1.2 [55]. The trimmed alignment was used for tree inference by RAxML-NG v.0.9.0 using default settings (Additional file 5) [56] and the best amino acid substitution model WAG + I + G4 + F (according to AIC), as determined by ModelTest-NG [57]. Bootstrap trees were calculated until their average weighted Robinson-Foulds distance dropped below the default cut-off of 3% for > 990/1000 permutations, resulting in 360 bootstrap replications [58].

### Construction of the expression vectors and golden gate cloning

Selected CDH III sequences were synthesized as linear gene fragments obtained from Twist Bioscience (https://www.twistbioscience.com/) with a length from 300 to 900 bp per fragment. Gene sequences were modified to meet the requirements for the subsequent cloning strategy involving (i) definition of gene sections of appropriate length, (ii) dividing the genes, into SP, Cyt and DH, (iii) removal of specific restriction sites (*Sac*I, *Pme*I, *Asc*I, *Bbs*I, *Bsa*I) by the introduction of synonymous codons, (iv) removal of the stop codon and (v) introduction of flanking regions for the golden gate cloning reaction. CDH sequences were divided within the linker region between the Cyt and the DH domain. Wherever possible, this was done eight residues before the conserved YDY-motif at the start of the Rossman fold (Fig. 2). As the SP fragment length was consistently below 300 bp, spacers were incorporated for gene fragment synthesis, which were subsequently removed in the golden gate reaction.

For propagation and subsequent cloning, linear fragments were inserted in the shuttle plasmid pAGM9121 and transformed in electrocompetent *E. coli* NEB 5α cells by electroporation using a MicroPulser Electroporator (Bio-Rad). The integration was achieved using 1 U µL^− 1 ^*Bbs*I-HF and 40 U µL^− 1^ T4 DNA ligase for 2 h at 37 °C, followed by an inactivation step at 80 °C for 20 min. *E. coli* transformation was performed by electroporation, using a MicroPulser Electroporator (Bio-Rad). Selection on spectinomycin-containing plates as well as a blue-white colony reporter system were used for the identification of clones carrying the desired plasmid. Plasmid DNA was extracted using a Monarch Plasmid Miniprep Kit (NEB). Purified donor plasmids, carrying the different modules (SP, Cyt, DH and an additional C-terminal fragment containing the TAA stop codon), were mixed in equal ratios in a one-pot reaction for the insertion into an acceptor plasmid, containing either the constitutive GAP promoter (pPAP009), or the methanol-induced AOX promoter (pPAP010). The ligation reaction was performed using 1.2 U µl^− 1^ *Bsa*I-HFv2 restriction enzyme and 40 U µl^− 1^ T4 DNA ligase in a thermocycler for a total of 60 cycles of 5 min at 37 °C, followed by 5 min at 16 °C and a final inactivation step of 20 min at 80 °C.

The resulting reaction mixture was used for the transformation of electrocompetent *E. coli* NEB 5α. By selection on kanamycin containing plates and blue-white screening, recombinant clones were selected and correct integration was verified for each clone by plasmid DNA sequencing. Vector maps for all plasmids used are depicted in Additional file 1: Figure S5. Plasmids were linearized by *Asc*I and transformed by electroporation into electrocompetent *K. phaffii* cells using a MicroPulser system at 1.5 kV and 3 ms. Multiple yeast clones containing the complete gene of interest integrated in its genome were obtained and selected by cultivation on selective hygromycin-containing YPD plates.

### Shake flask expression and electrophoretic analysis

Initial expression screening was performed by cultivation of *K. phaffii* transformants harboring CDH III encoding genes with their native SP under the control of the GAP promoter in shake flasks. Cells were grown at 30 °C for a total of 6 days and glucose was fed to the culture with a final concentration of 2% four days after inoculation. To evaluate the expression of the target proteins, SDS-PAGE was performed using 4–20% Mini-PROTEAN TGX Stain-Free precast gels (Bio-Rad) following the manufacturer’s instructions and using Precision Plus Protein Standard (Bio-Rad). The samples of the culture supernatant were deglycosylated with Endo H_f_ (NEB) under denaturating conditions according to manufacturer’s instructions.

### Signal peptide shuffling and microtiter plate screening

An adapted version of the procedure described by Püllmann et al. was employed to screen for improved expression using different SP sequences and promoters [32]. Briefly, the construction of the final integration plasmid, containing the gene of *F. solani* CDH III (KAH7237211.1), was performed incorporating an equal ratio mixture of ten different signal peptides inserted in donor plasmids (Additional file 1: Table S4). Two SP sequences of the putative CDH-encoding genes from *F. solani* and eight different SP sequences obtained from Addgene (Kit #1,000,000,166) were used for this purpose. Two reactions were set up: one using the pPAP009 acceptor integration plasmid containing the GAP promoter, and the second using the pPAP010 acceptor integration plasmid containing the AOX promoter (Additional file 1: Fig. S5). The two resulting plasmid libraries were used as previously described in the result and discussion section for propagation in *E. coli* NEB 5α and subsequently for the transformation of *K. phaffii*.

The expression screening was performed in 96-deep-well-plates and single colonies were used for the inoculation of 250 µl BYPD supplemented with 150 mg L^− 1^ hygromycin. Plates were sealed with oxygen permeable Breathe-Easy sealing membranes (Diversified Biotech) and incubated at 30 °C, 350 rpm and 80% humidity. For plates with clones of the pPAP010 library, containing the AOX promoter, expression was induced after 65 h by adding 250 µl of BMMY medium, consisting of 1% yeast extract, 2% peptone, 100 mM potassium phosphate pH 6.0, 1.34% Yeast Nitrogen Base (YNB), 4 × 10^− 5^% biotin, 0.5% methanol. A feeding of 50 µl of 5% (v/v) MeOH for was supplied after 8, 24, and 48 h of incubation and the supernatant was harvested after a total time of 137 h. For plates with clones of the pPAP009 library, containing the GAP promoter, no further feeding was performed, and the supernatant was harvested after 72 h of cultivation.

Cells were removed by centrifugation, CDH activity in the supernatant was measured using the standard DCIP (ε_520_ = 6.9 mM^− 1^ cm^− 1^) assay conditions: 50 mM potassium phosphate buffer pH 6.0, containing 0.3 mM DCIP and 1 mM cellobiose as substrate. The absorbance at 520 nm was recorded using an EnSpire Multimode plate reader (PerkinElmer) for 20 min. The limit of quantification was calculated as 10 times the standard deviation obtained by the measurement of blank DCIP reactions without the addition of cellobiose.

Clones with the highest activities in the supernatant were analyzed by colony PCR to verify gene integration and identify the respective SP responsible for high activity expression. Genomic DNA was isolated following a published procedure [59] and the gene of interest was amplified by PCR using OneTaq polymerase (NEB) according to the procedure described by the manufacturer with 5_AOX_fwd, positioned in the AOX promoter region, and 3_H_rev located in the *Fs*CDH encoding ORF as primers (Additional file 1: Table S5). Amplified DNA was purified using a PCR & DNA Cleanup Kit (NEB) and the identity of the obtained PCR product, harboring the signal peptide, was analyzed by sequencing (Microsynth).

### Recombinant production *Fs*CDH

The protocol for the large-scale production of *Fs*CDH in *K. phaffii* was adapted from the Pichia Fermentation Process Guidelines (Invitrogen) and carried out in a BioFlo 120 (Eppendorf) bioreactor. The inoculum (400 mL) for the bioreactor was grown in two 200 mL shake flasks cultures for 18 h at 30° C and 115 rpm in BYPD and was subsequently used to inoculate 3.2 L of basal salt medium containing 4% (w/v) glycerol as the carbon source and supplemented with 4.35 mL L^− 1^ Pichia trace metal solution (PTM_1_). The temperature was maintained at 30 °C and the pH was constantly regulated to 5.0 by adding 25% (w/v) ammonium hydroxide. The aeration was set to 1.0 vvm throughout the whole fermentation. During the glycerol batch phase, an agitation speed of 750 rpm was used. For the following glycerol fed batch phase, a feed of 300 mL glycerol 50% (w/v), supplemented with 12 mL L^− 1^ PTM_1_, was initiated and the feed rate was adjusted to keep the oxygen level at 40%. After 24 h, methanol was added to a final concentration of 0.5% (v/v) to induce expression. Protein expression was carried out with a feed of 100% methanol, containing 12 mL L^− 1^ PTM_1_, and a feed rate set to maintain 20% oxygen saturation. The progress of the fermentation process was monitored by measuring wet biomass, protein concentration, and enzyme activity. The latter was measured by two standard assays: the previously described DCIP assay and the cytochrome *c* assay (ε_550_ = 19.6 mM^− 1^ cm^− 1^). This last standard reaction mix consisted of 20 µM cytochrome *c*, 50 mM potassium phosphate buffer at pH 6.0 and 1 mM of substrates. Absorbance was recorded at 550 nm. Both assays were performed at 30 °C and the change in absorbance at the respective wavelength was measured for 180 s in a Lambda 35 UV/Vis Spectrometer (PerkinElmer). The fermentation was stopped after 71 h from initial methanol induction and 95 h after inoculation The supernatant was collected after pelleting the cells by centrifugation at 7900 x g and 4 °C for 20 min.

### Purification of *Fs*CDH

To isolate the recombinantly expressed protein, a two-step chromatographic purification strategy was employed. As a first capturing step, hydrophobic interaction chromatography (HIC) was used with a 500 ml Phenyl-Sepharose Fast Flow column (Cytiva), equilibrated with 50 mM sodium acetate, adjusted to pH 5.5 and containing 30% (sat.) ammonium sulfate. Elution was carried out in a linear gradient over 3 column volumes, to 0% (sat.) ammonium sulfate, with the same buffer. Fractions were pooled based on their specific activity and desalted with a Vivaflow® 50 crossflow module (Sartorius) with a cut-off of 30 kDa, resulting in two main pools corresponding to the full-length enzyme and the cleaved DH domain. The second purification step was carried out by anion exchange chromatography (AEX), using a 52 ml SOURCE 30Q (Cytiva) equilibrated with 10 mM HEPES buffer, pH 7.0. Elution was performed in a linear gradient from 0 to 0.5 M NaCl over 10 column volumes using the same buffer. Fractions of the full-length enzyme or the DH domain were pooled according to their specific activity and RZ values (A_420_/A_280_). The enzyme preparations were finally desalted, concentrated and rebuffered to 10 mM HEPES buffer pH 7.0 using Amicon Ultra Centrifugal Filters (Merck Millipore) with a 30 kDa cut-off. Aliquots of purified *Fs*CDH were stored at -80 °C. SDS-PAGE analysis, as described above, was used to evaluate the purity, to estimate the degree of glycosylation and to determine the molar masses of the obtained enzymatic fractions.

### Activity assays and spectrophotometric analysis

One unit of enzyme activity was defined as the amount of enzyme capable of oxidizing 1 µmol of the electron acceptor per minute under the given conditions. Spectrophotometric assays were performed on a Lambda 35 UV/Vis Spectrometer (PerkinElmer), employing either cytochrome *c* for full-length CDH, or DCIP for both, the full-length CDH and the DH domain, as electron acceptors. Enzyme activity was determined using the standard assays conditions as described above, monitoring the activity in buffer systems adjusted to three different pH values: pH 4.0 in 50 mM sodium acetate buffer, pH 6.0 in 50 mM potassium phosphate buffer and pH 8.0 in 50 mM HEPES buffer. As regards DCIP, absorbance was recorded at 520 nm for pH 4.0 and 6.0, and at 600 nm for pH 8.0 (DCIP ε_600_ = 11.8 mM^− 1^ cm^− 1^). The concentration of both cellobiose and lactose was 1 mM.

Spectra of purified proteins were recorded at 21 °C in both oxidized and reduced states, using an Agilent 8453 UV-Vis Spectrophotometer (Agilent). Protein samples of the full-length enzyme and the isolated DH domain in the oxidized state, were diluted in 10 mM HEPES buffer, pH 7.0, to an absorbance at 280 nm of ~ 1.5. Spectra were recorded before and after the addition of 1 mM cellobiose and incubation for 10 min. Molar absorption coefficients at 280 nm were determined from the mature amino acid sequence using ProtParam (http://web.expasy.org/protparam/) with values of 100.2 mM^− 1^ cm^− 1^ for the full-length CDH and 77.6 mM^− 1^ cm^− 1^ for the DH domain and were used for the determination of the protein concentration of the purified enzymes. The purity of the different enzyme preparations was further evaluated based on RZ values, defined as the ratio of A_420_ to A_280_.

FAD occupancy was determined for *Fs*CDH, *Fs*DH and *Mt*CDH using the TCA method [60]. This method involved the addition of 5% (for *Fs*CDH and *Mt*CDH) and 20% (for *Fs*DH) trichloroacetic acid to release and spectrophotometrically measure the non-covalently bound FAD cofactor. FAD loading was iteratively calculated following established protocols [34].

### Electronic supplementary material

Below is the link to the electronic supplementary material.

Additional file 1. Table S1. List of 14 characterized CDHs. Table S2. List of 17 characterized GMCs. Fig. S1. Sequence similarity network at an alignment score cut-off of 10^– 40^. Fig. S2. Sequence similarity network at an alignment score cut-off of 10^– 140^. Fig. S3. Sequence similarity network at an alignment score cut-off of 10^– 160^. Table S3. Selected CDH III sequences for heterologous expression with phylogenetic clade labels as defined in Fig. 3. Fig. S4. SDS-PAGE of crude extracts derived from the culture supernatant of preliminary experiment in shake flasks tested for class III CDH production. Fig. S5. Plasmid map of the vectors used for golden gate cloning and signal peptide shuffling. Table S4. List of used signal peptide basic modules for signal peptide shuffling. Table S5. Primers used for colony PCR.

Additional file 2. FASTA input file for SSN calculation submitted to the EFI-EST web tool, consisting of 5369 sequences, from two database (NCBI and Uniprot) searches, 14 characterized CDHs and 17 characterized non-CDH GMC enzymes.

Additional file 3. FASTA file of all sequences extracted from the CDH class III cluster.

Additional file 4. FASTA file of sequences used for inferring the phylogenetic tree.

Additional file 5. Newick file of the maximum likelihood tree for CDH class III.

## Data Availability

All data generated or analyzed in the course of this study has been included in the manuscript or supplementary information files.
