# Supplementary material for: Exploring class III cellobiose dehydrogenase: sequence analysis and optimized recombinant expression
Source: Microb Cell Fact. 2024 May 23;23:146. doi: 10.1186/s12934-024-02420-2 (PMC11112829; doi:10.1186/s12934-024-02420-2)
Supplement: Supplementary file 1 — Additional file 1. Table S1. List of 14 characterized CDHs. Table S2. List of 17 characterized GMCs. Fig. S1. Sequence similarity network at an alignment score cut-off of 10– 40. Fig. S2. Sequence similarity network at an alignment score cut-off of 10– 140. Fig. S3. Sequence similarity network at an alignment score cut-off of 10– 160. Table S3. Selected CDH III sequences for heterologous expression with phylogenetic clade labels as defined in Fig. 3. Fig. S4. SDS-PAGE of crude extracts derived from the culture supernatant of preliminary experiment in shake flasks tested for class III CDH production. Fig. S5. Plasmid map of the vectors used for golden gate cloning and signal peptide shuffling. Table S4. List of used signal peptide basic modules for signal peptide shuffling. Table S5. Primers used for colony PCR. [file 12934_2024_2420_MOESM1_ESM.pdf]

Supplementary Information

Exploring class III cellobiose dehydrogenase: Sequence analysis and optimized recombinant expression

Angela Giorgianni, Alice Zenone, Leander Sützl, Florian Csarman*

and Roland Ludwig

BOKU University, Department of Food Science and Technology, Institute of Food Technology, Muthgasse 18, 1190 Vienna, Austria

Table S1 List of characterized CDHs. Amino acid sequences were used as inputs for NCBI and Uniprot databases searches.

UniProt ID	Organism	CDH class	Length (aa)	Reference
Q01738	*Phanerodontia chrysosporium* (syn. *Phanerochaete chrysosporium*)	I	773	(26)
A9XK88	*Thermothelomyces myriococcoides* (syn. *Crassicarpon hotsonii,* syn. *Myriococcum thermophilum*)	II	828	(27)
Q7RXM0	*Neurospora crassa*	II	829	(4,28)
B6D1P2	*Gelatoporia subvermispora*	I	774	(29)
Q9P8H5	*Humicola insolens*	II	785	(30)
O74253	*Trametes cinnabarina* (*syn. Pycnoporus cinnabarinus*)	I	769	(31)
Q875J3	*Trametes versicolor*	I	768	(32)
E7D6C0	*Amesia atrobrunnea*	II	831	(4)
E7D6B9	*Thermothelomyces fergusii* (*syn. Crassicarpon thermophilum,* syn. *Corynascus thermophilus*)	II	787	(4)
E7D6C1	*Dichomera saubinetii*	II	837	(4)
E7D6C3	*Hypoxylon haematostroma*	II	802	(4)
Q7S0Y1	*Neurospora crassa*	II	828	(28)
E7D6C5	*Stachybotrys bisbyi*	II	832	(4)
A0A219WGI5	*Termitomyces clypeatus*	n.d.	840	(33)

Table S2 List of characterized GMCs. Amino acid sequences were used as outgroup for the construction of the sequence similarity network.

UniProt ID	PDB	GMC	Organism	Length	Reference
Q5B2E9	-	Pyranose 2-oxidase (POx)	*Emericella nidulans*	601	(34)
Q8J2V8	-	Pyranose 2-oxidase (POx)	*Tricholoma matsutake*	564	(35)
Q6QWR1	4MIF	Pyranose 2-oxidase (POx)	*Phanerodontia chrysosporium*	621	(36)
V5NDL4	-	Pyranose dehydrogenase (PDH)	*Agaricus campestris*	595	(37)
Q3L1D1	-	Pyranose dehydrogenase (PDH)	*Agaricus bisporus*	594	(38)
Q3L245	4H7U	Pyranose dehydrogenase (PDH)	*Leucoagaricus meleagris*	602	(39)
Q92452	-	Glucose oxidase (GOX)	*Talaromyces flavus*	605	(40)
P81156	1GPE	Glucose oxidase (GOX)	*Penicillium amagasakiense*	587	(41)
P13006	1CF3	Glucose oxidase (GOX)	*Aspergillus niger*	605	(41)
B8MX95	4NYU	Glucose oxidase (GOX)	*Aspergillus flavus*	593	(40)
G8E4B4	-	Glucose dehydrogenase (GDH)	*Colletotrichum gloeosporioides*	600	(42)
I7FDJ2	-	Alcohol oxidase (AOx)	*Aspergillus terreus*	666	(43)
F2QY27	5HSA	Alcohol oxidase (AOx)	*Komagataella phaffii*	663	(44)
A8QJP8	-	Alcohol oxidase (AOx)	*Moniliophthora perniciosa*	378	(45)
A0A060SUP1	-	Aryl-alcohol oxidase (AAO)	*Pycnoporus cinnabarinus*	554	(46)
A0A164NR05	-	Aryl-alcohol oxidase (AAO)	*Sistotremastrum niveocremeum*	596	(47)
O94219	3FIM	Aryl-alcohol oxidase (AAO)	*Pleurotus eryngii*	593	(48)

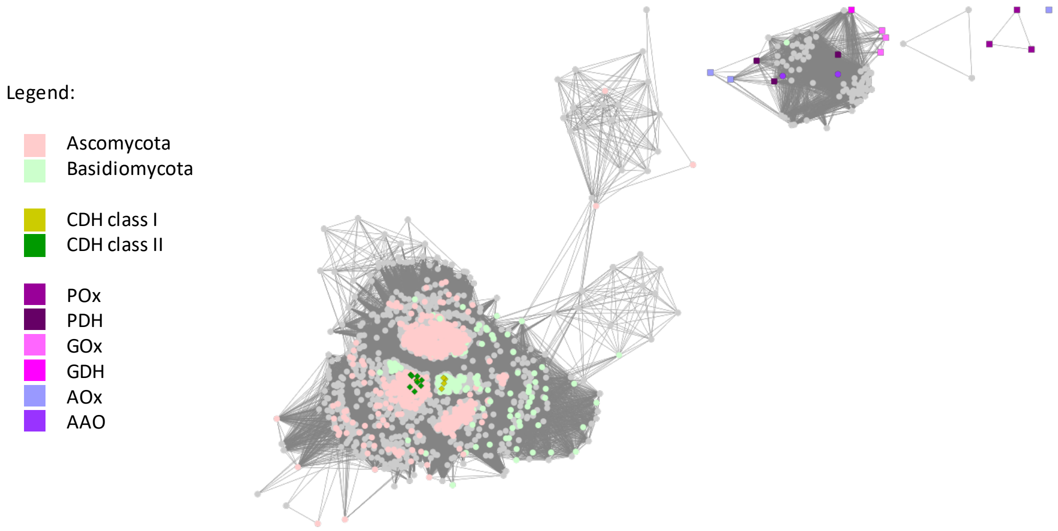

Fig. S1 Sequence similarity network at an alignment score cut-off of 10^{-40} showing the separation of the GMC-oxidoreductase outgroup.

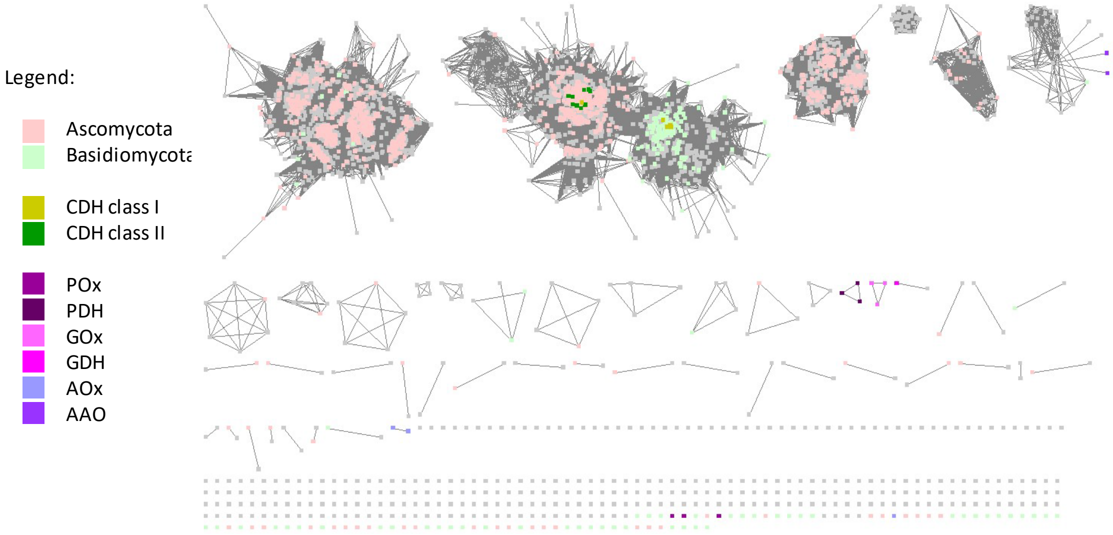

Fig. S2 Sequence similarity network at an alignment score cut-off of 10^{-140} showing the separation of class III CDHs.

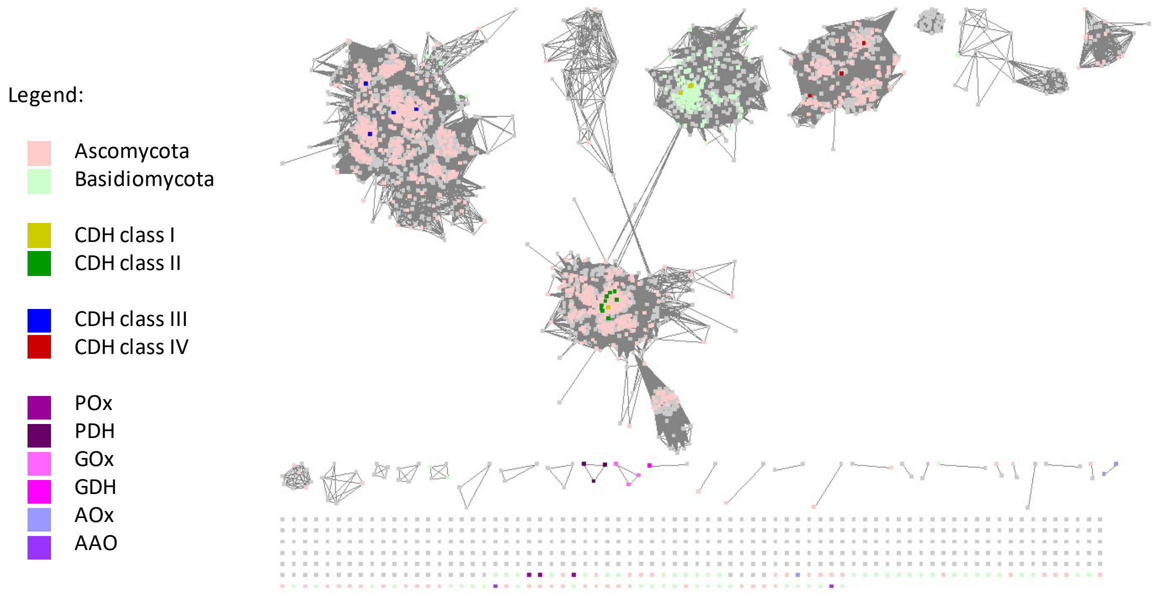

Fig. S3 Sequence similarity network at an alignment score cut-off of 10^{-160} showing the separation between class I and II CDHs.

Table S3 Selected CDH Sequences for heterologous production with phylogenetic clade labels as defined in Figure 3.

Phylogenetic tree clade	Database ID	Originating species	Length (aa)
1a	A0A2P5IFS2	*Diaporthe helianthi*	770
1a	A0A194V299	*Valsa mali*	800
1c	KAF2998525_1	*Curvularia kusanoi*	923
1c	A0A2V1DC04	*Periconia macrospinosa*	889
1a	KAH7237211.1	*Fusarium solani*	799
1b	A0A218Z990	*Marssonina coronariae*	774
1c	KAF1997296.1	*Amniculicola lignicola*	758
2	KAG2163296.1	*Aureobasidium pullulans*	879
3	RDI76702.1	*Venturia inaequalis*	770
4b	XP_025427267.1	*Aspergillus saccharolyticus*	912
4c	KAH7254564.1	*Fusarium solani*	802

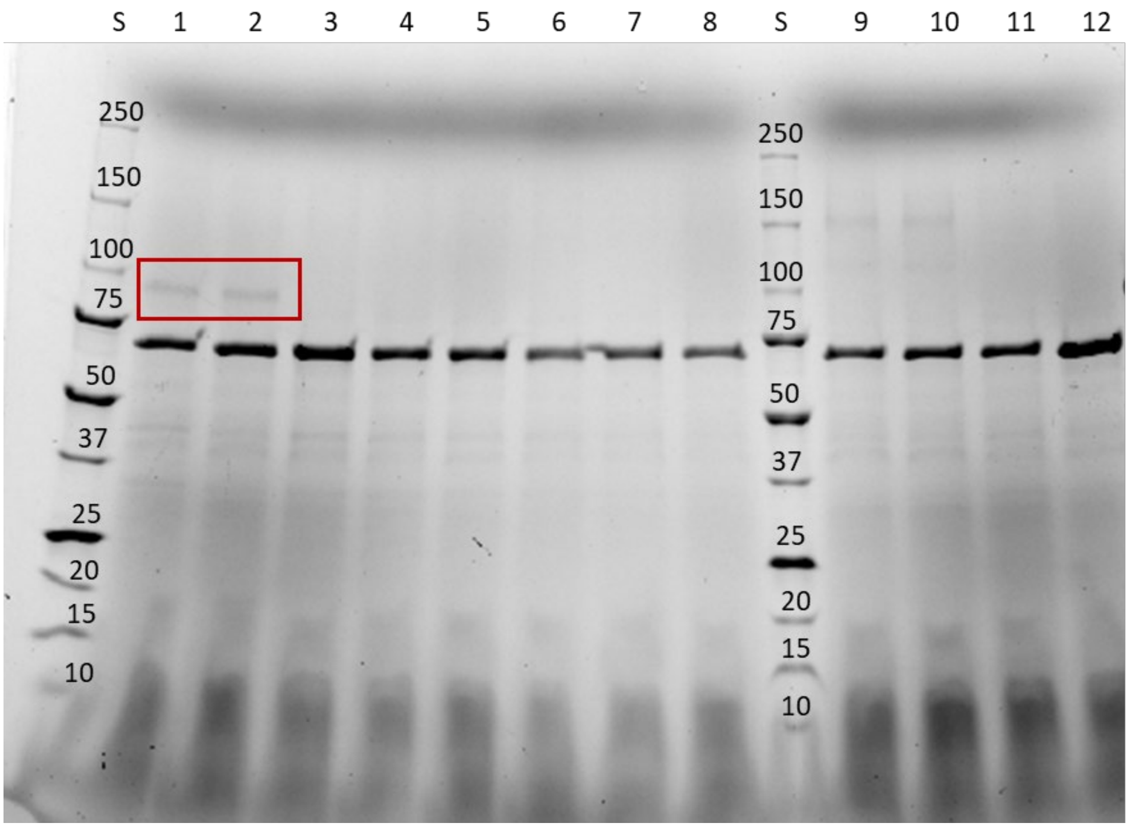

Fig.S4 SDS-PAGE of crude extracts derived from the culture supernatant of preliminary experiment in shake flasks tested for class III CDH production. Deglycosylation treatment with Endo-Hf (NEB, molecular weight ~70 kDa) was performed prior to SDS-PAGE analysis. Precision Plus Protein Dual Color Standard (Bio-Rad) is shown in Lane S. Lane 1 and 2 show *Fs*CDH (in a red box), Lane 3 and 4 CDHIII from *Marssonina coronarie*, Lane 5 and 6 CDHIII from *Aureobasidium pullulans*, Lane 7 and 8 CDHIII from *Venturia inaequalis*, Lane 9 and 10 CDHIII from *Aspergillus saccharolyticus* and Lane 11 and 12 a second CDHIII from *F. solani* (KAH7254564.1).

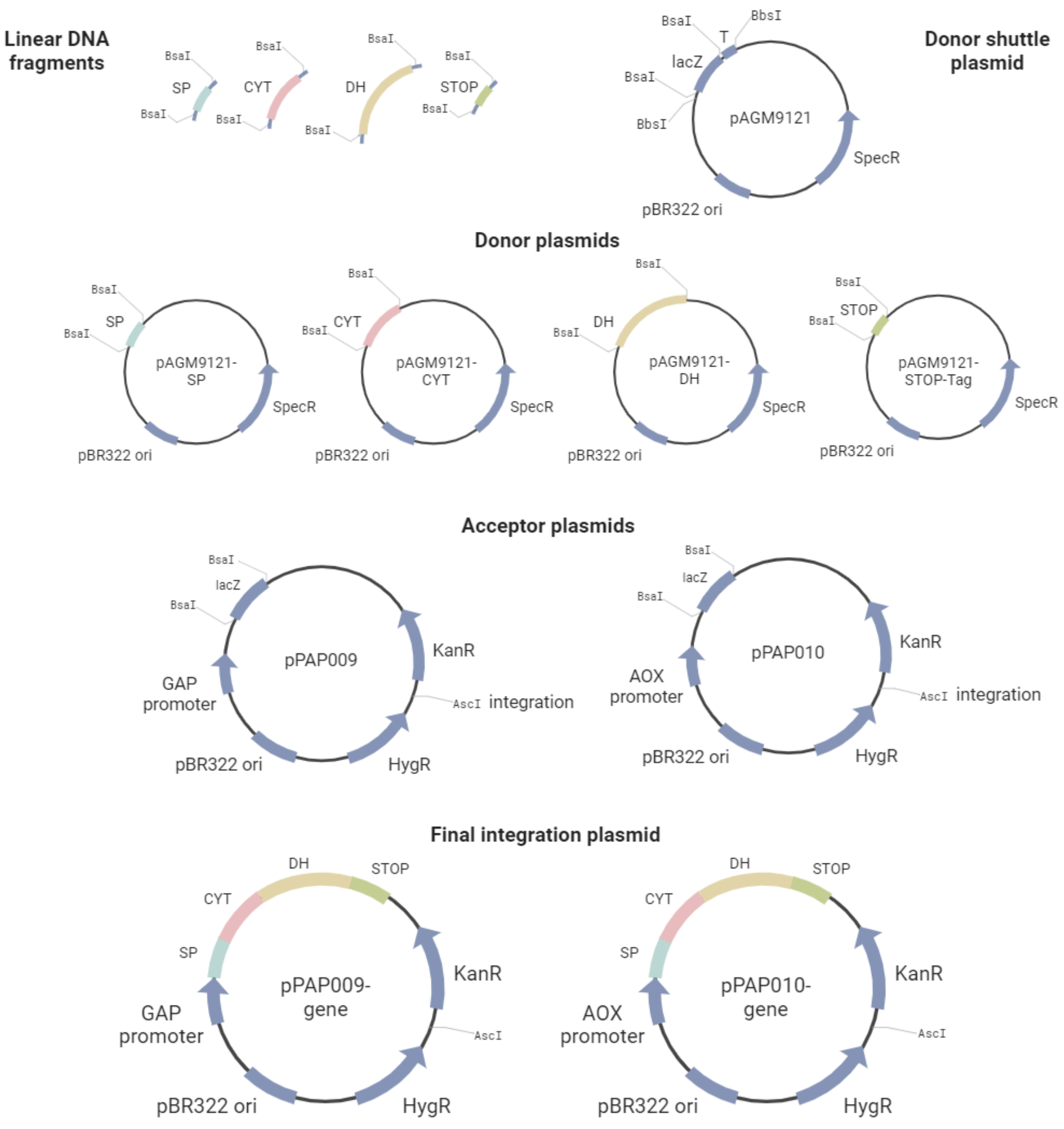

Fig.S5 Plasmid map of the vectors used for golden gate cloning and signal peptide shuffling. Linear cDNA fragments corresponding to signal peptide (SP, turquois), cytochrome domain (CYT, red), dehydrogenase domain (DH, yellow) and Stop-Tag (STOP, green) were inserted in the donor shuttle plasmid pAGM9121. After restriction/ligation reaction, transformation and isolation of plasmidic DNA, donor plasmids were obtained, in which every fragment was singularly integrated into pAGM9121. These plasmids were then mixed in equal ratio to a one-pot final reaction for the insertion into an acceptor plasmid for genomic integration that already contains either the constitutive GAP promoter, pPAP009, or the methanol-induced AOX promoter, pPAP010. After restriction/ligation reaction, transformation and isolation of plasmidic DNA, final expression vectors for the genomic integration in the expression yeast host were obtained. Figure created with BioRender.com

Table S4 List of used signal peptide basic modules for signal peptide shuffling.

Name	Source	cDNA inserted in	Lenght (aa)	Amino acid sequence
*Fs*CDH_SP	CDH from *F. solani* (ID KAH7237211.1)	pAGM9121	20	MQFKFLLSSAALLLTGFAQA
*Fs*CDH2_SP	CDH from *F. solani* (ID KAH7254564.1)	pAGM9121	20	MRFNLSSMATLLGLVGTTTA
Sce-Prepro SP (α factor)	α-mating factor from *Saccharomyces cerevisiae*	pAGM9121	87	MRFPSIFTAVLFAASSALAAPVNTTTEDE TAQIPAEAVIGYLDLEGDFDVAVLPFSNS TNNGLLFINTTIASIAAKEEGVSLDKREA
Kma-Inulinase SP	Inulinase from *Kluyveromyces marxianus*	pAGM9121	15	MKLAYSLLLPLAGVS
Sce-Invertase 2 SP	Invertase from *S. cerevisiae*	pAGM9121	17	MLLQAFLFLLAGFAAKI
Sce-Acid Phosphatase SP	Acid phosphatase from *S. cerevisiae*	pAGM9121	16	MFKSVVYSILAASLAN
Hsa-Serum Albumin SP	Human serum albumin	pAGM9121	17	MKWVTFISLLFLFSSAY
Sce-Killer Protein SP	Killer protein from *S. cerevisiae*	pAGM9121	24	MTKPTQVLVRSVSILFFITLLHLV
Ani-α Amylase SP	α-Amylase from *Aspergillus niger*	pAGM9121	18	MVAWWSLFLYGLQVAAPA
Sce-α Galactosidase SP	α-Galactosidase from *S. cerevisiae*	pAGM9121	17	MFAFYFLTACISLKGVF

Table S5. Primers used for colony PCR.

Primer name	Primer sequence (5' to 3')
5_AOX_fwd	5-GACTGGTTCCAATTGACAAGC-3
3_FsCDH_rev	5-AGAATTCCGGGAGAGTCAGAG-3
